# Optimization of High-Q Coupled Nanobeam Cavity for Label-Free Sensing

**DOI:** 10.3390/s151025868

**Published:** 2015-10-13

**Authors:** Mohammad Tariq Yaseen, Yi-Chun Yang, Min-Hsiung Shih, Yia-Chung Chang

**Affiliations:** 1 Department of Engineering and System Science, National Tsing Hua University, Hsinchu 300, Taiwan; E-Mail: mtyaseen@yahoo.com; 2 Nano Science and Technology Program, Taiwan International Graduate Program, Academia Sinica, Taipei 115, Taiwan; 3 Research Centre for Applied Sciences, Academia Sinica, Taipei 115, Taiwan; E-Mail: tigpnano2008@gmail.com; 4 Department of Physics, National Cheng Kung University, Tainan 701, Taiwan; 5 Department of Photonics, National Chiao Tung University, Hsinchu 300, Taiwan; 6 Department of Photonics, National Sun Yat-sen University, Kaohsiung 80424, Taiwan

**Keywords:** Photonic crystals (PhCs), nanobeam (NB) cavity, finite-difference time-domain (FDTD), label-free sensing

## Abstract

We numerically and experimentally investigated the lateral coupling between photonic crystal (PhC) nanobeam (NB) cavities, pursuing high sensitivity and figure of merit (FOM) label-free biosensor. We numerically carried out 3D finite-difference time-domain (3D-FDTD) and the finite element method (FEM) simulations. We showed that when two PhC NB cavities separated by a small gap are evanescently coupled, the variation in the gap width significantly changes the coupling efficiency between the two coupled NB cavities and the resulting resonant frequencies split. Experimentally, we fabricated laterally-coupled PhC NB cavities using (InGaAsP) layer on the InP substrate. For sensing, we showed that the laterally coupled PhC NB cavities sensor exhibits higher sensitivity than the single PhC NB cavity. The higher sensitivity of laterally coupled PhC NB cavities is due to the strong evanescent coupling between nearby PhC NB cavities, which depends on the gap width and it is attributed to the large confinement of the electromagnetic field in the gap (air or liquid). As a result of the lateral coupling, both even (symmetric) and odd (asymmetric) modes exist. We show that even modes are more sensitive than odd modes. In addition, higher-order modes exhibit higher sensitivity. Hence, we characterized and examined the fabricated PhC NB cavity as a label-free biosensor, and it exhibits high figure of merit due to its high Q-factor. This illustrates a potentially useful method for optical sensing at nanoscale.

## 1. Introduction

Optical biosensing based on photonic devices such as interferometers [1,2], resonators [3,4], plasmonic structures [5,6], slot waveguides [7,8], and photonic crystals (PhC) [9,10,11,12] has attracted much attention in recent years for lab-on-chip bio applications. PhC nanocavities have attracted significant interest recently for their ability to confine light strongly in wavelength-scale volumes. The high-Q factors, small mode volumes (of the order of optical wavelength [~(λ*/n*)*^x^*], where λ is the resonant wavelength in vacuum, *n* is the refractive index of the slab, and the exponent *x* depends on the design itself.), highly compact and integrative properties of these PhC nanocavities have allowed a wealth of new applications in sensing [13,14,15,16].The sensing concept of these photonic devices is based on the electromagnetic field overlapping with the surrounding medium, which allows label-free detection of the refractive index change. Due to the low cost and label-free aspects, this type of biosensors may strongly affect cellular sensing and biochemical recognition [17,18].

However, with the high-Q PhC cavity optical fields are mainly concentrated in the high index medium, which leads to the limited sensitivity. It cannot efficiently sense the change in the refractive index of surrounding medium. Therefore, the sensitivity is very low due to the weak overlapping between the electromagnetic field and the refractive index of surrounding medium [19,20,21,22,23,24,25,26]. To overcome this limitation, light should be confined outside the high index material. For instance, it can be confined in air instead of the dielectric to form an air-slot cavity; this cavity is more sensitive to change in the surrounding medium’s refractive index because of the strong overlapping between the electromagnetic field and the surrounding medium [15,27]. 

The overlap of the surrounding medium with the evanescent tail of the propagating optical field in the PhC cavity can lead to a detectable spectral shift in the cavity resonance wavelength when the refractive index of the surrounding changes. High Q-factor leads to much narrower line-width and improves the figure of merit (FOM). The FOM of sensors is defined as (*S∙Q/*λ*_res_*), where S is the sensitivity (the amount of wavelength shift due to the change of the refractive index of the surrounding medium), Q is the quality factor, and λres is the resonance wavelength [19,20,21,22,23,24,25,26].The FOM is linearly proportional to the Q-factor. High Q-factor improves the wavelength resolution, and as a result strongly enhances the sensor performance [25,28]. 

Putting two identical PhC nanobeam (NB) cavities in parallel to each other will produce more extended modes due to coupling effect. These modes have even and odd profiles with frequency splitting (frequencies shifted up and down from the level of single NB cavity). The frequency splitting between the modes is indirectly proportional to the gap value between the two PhC NB cavities [29,30,31,32,33,34,35,36,37]. The laterally-coupled PhC NB cavities can tune the optical field profile in between the cavities at nanoscale. Additionally, operating wavelength shift of the PhC NB cavity due to the coupling is potentially beneficial for various applications including biosensing [38,39]. Small-scale optical sensors such as ring resonators are also very promising for biosensing because of their many advantages which include, for example, fast optical response, low loss, label-free detection, good compatibility, and integration with microfluidic channels [40,41,42]. In this work, a laterally-coupled PhC NB cavity was investigated to pursue biosensing of high sensitivity and FOM. The laterally-coupled PhC NB cavity that provides small mode volume [*Vmod ~* *(*λ*/n)^3^*], ultra-high Q-factor (>10^6^) and high (*Q/Vmod*), yields an excellent sensing performance.

## 2. Numerical Modeling

### 2.1. Single PhC NB Cavity

**Figure 1:**
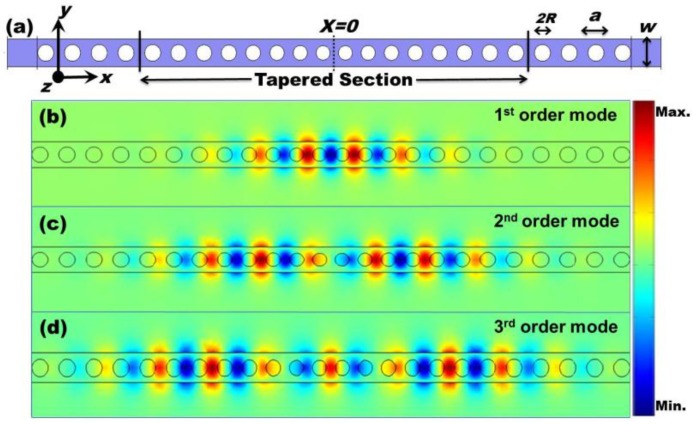
(**a**) Schematic of the single PhC NB cavity; (**b**–**d**) mode profiles of the electric field components Ey for the first, second, and third order mode, respectively.

The single PhC NB has several holes separated by a lattice constant (a) making it function as a Bragg reflector that confines light along the axis of the PhC NB, which is the *x*-axis as shown in Figure 1a, and leads to a band gap of the PhC device. However, tapering the holes near the center (*x* = 0) of the PhC NB device introduces some modes in the band gap. Particular modes are supported in that region of defect because of reduced mismatch; hence, high Q-factor modes exist. On the other hand, the ends of this PhC device, in which the holes are not tapered, serve as reflecting mirrors that cause the PhC NB device to become a cavity. As a result, resonant modes exist, as shown in Figure 1. Hence, the coupling between the two PhC NB cavities was investigated as a method of enhancing the sensitivity of the single PhC NB cavity. The sensitivity of the single PhC NB cavity acts as a reference for the enhancement by shifting the electromagnetic field outside the dielectric to increase overlapping area with the surrounding medium, making it sensitive to any minute shift in resonant wavelength for refractive index sensing. Sensitivity thus increases, while preserving an ultra-high Q-factor (very narrow line-width).

### 2.2. Coupled PhC NB Cavity

**Figure 2:**
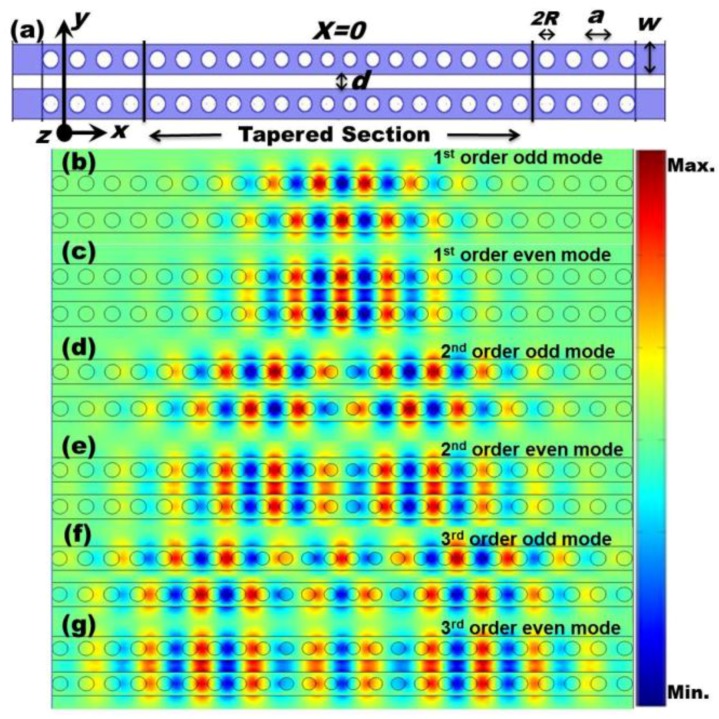
(**a**) Schematic of the investigated biosensor based on the laterally coupled PhC NB cavities (*d* = 0.5*a*); (**b**–**g**) mode profiles of the electric field components *E*_y_ for odd and even modes derived from first, second, and third order modes, respectively.

Figure 2a shows the schematic of a laterally-coupled PhC NB cavity of concern, which involves two identical single PhC NB cavities. The single PhC NB cavity, as shown in Figure 1a, operates near (1.5 μm) with a high Q-factor (Q > 10^6^). In this single PhC NB cavity, light is confined by an index guiding in the *y* and *z* directions and Bragg reflection in the *x* direction, as shown in Figure 1b–d. However, the coupled PhC NB cavity design is based on using lateral coupling between these two PhC NB cavities, where the two parallel, freestanding PhC NB cavities are separated by an air gap width (*d*), as shown in Figure 2a. Due to the evanescent coupling effect, the electromagnetic field extends to the gap (low refractive index material) between the two PhC NB cavities. For this structure, the Q-factor and the resonant wavelength (λ) are highly sensitive to the variation of the gap width (*d*) and the refractive index of the surrounding medium. Each investigated PhC NB cavity, as shown in Figure 1a and Figure 2a, is composed of a suspended (freestanding) PhC slab with an array of air holes of lattice constant (*a*) and radius (*R*), in which the middle section is replaced by a tapered structure with 16 tapered air holes. The radii of holes and center-to-center distances are gradually reduced toward the center of the cavity, forming a cavity sandwiched between the two PhC Bragg mirrors. Thus, the tapered design can reduce modal mismatch of the electromagnetic field entering from the center into the mirror region to increase light localization around the center. 

In the simulation of the PhC NB cavity, as shown in Figure 1a, the refractive index of the dielectric medium (InGaAsP layer), lattice constant, slab thickness, width, and air hole radius used are *n* = 3.4, *a* = 450 nm, *t* = 240 nm, *w* = 450 nm, and *R* = 135 nm, respectively. The eight air holes (*R*1–*R*8) of the right half of the tapered section have radii are 0.252*a*, 0.258*a*, 0.264*a*, 0.270*a*, 0.276*a*, 0.282*a*, 0.288*a*, and 0.296*a*, and center-to-center distances of 0.84*a*, 0.86*a*, 0.88*a*, 0.90*a*, 0.92*a*, 0.94*a*, 0.96*a*, and 0.98*a*, respectively. PhCs have been proposed for optical sensing applications because of its ability to manipulate light by creating photonic band gaps through a periodic array of dielectric structures with size comparable to wavelength. Basically, sensing depends on overlapping between evanescent waves and the surrounding medium. It depends also on the Q-factor, which is a result of structural design and the material used in this design. For low Q-factor PhC structures and weak evanescence waves outside the structure, sensitivity would be very low and loss would probably be high. On another hand, for coupled PhC NB cavities, the Q-factor is high (>10^6^). In addition, the loss would be low because of its small cross-section. The lateral coupling between the PhC NB cavities extends the field outside the structures, especially for even modes, as shown in Figure 2c,e,g, that enhances the device sensitivity as a result of the overlapping increment between the field and the surrounding medium. For odd modes, most of the field is concentrated inside the dielectric structure, and therefore, less influenced by the change of surrounding medium index, and this would affect its performance as a sensor, as shown in Figure 2b,d,f. 

3D-FDTD simulation of the PhC NB cavity with subpixel averaging to increase accuracy was carried out. Several Gaussian sources with a center frequency and width matching that of the photonic band gap were positioned at different locations at the middle of the tapered section to excite available modes. However, harmonic inversion software based on the filter diagonalization method analyzes the resulting fields, which can extract decay rates and frequencies of the PhCNB cavity’s modes. In addition, we calculated the field profiles of the first three order modes (first, second, and third), as shown in Figure 1 and Figure 2, using finite-element method (FEM). The simulation results show that those three particular modes (first, second, and third) exist in both single and coupled PhC NB cavities, as shown in Figure 1 and Figure 2. In the simulation, the simulation domain was bordered by spatial-unit-thick perfectly matched layers (PMLs), which absorb the fields leaving the simulated region to prevent reflections. Such reflections could interfere with the measureable quantities of interest, namely cavity Q-factor and resonance frequency. 

**Figure 3:**
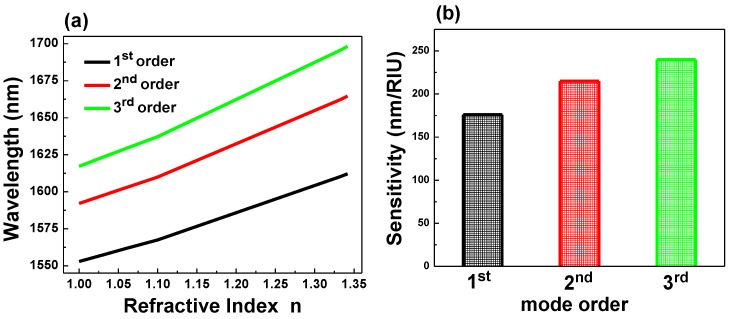
(**a**) Refractive index n *versus* wavelength shift ∆λ; (**b**) sensitivity (S = Δλ/Δ*n*) for (first, second, and third) modes for the single PhC cavity.

### 2.3. Sensitivity Calculation of the Single PhC NB Cavity

To calculate the refractive index sensitivity of the single PhC NB cavity for a comparison with the laterally coupled PhCNB cavity biosensor, we numerically simulated the first three modes (first, second, and third) that exist in the single PhC NB cavity, as shown in Figure 1b–d. Then we calculated the shift in cavity resonance in response to the variation of the surrounding medium’s refractive index; the result is shown in Figure 3a, in which the slope (S = Δλ/Δ*n*) of each illustrated curve represents the sensitivity of each particular mode of the first three modes, which is shown in Figure 3b. We found that the third-order mode has a larger wavelength shift than the first two modes. In other words, it has a higher refractive index sensitivity that is due to the field extension over a large area, as shown in Figure 1d. Hence, for sensing applications, it is desirable to design a device that has a large resonance frequency change for small variation in the surrounding refractive index medium.

### 2.4. Sensitivity Calculation of the Coupled PhC NB Cavity

**Figure 4:**
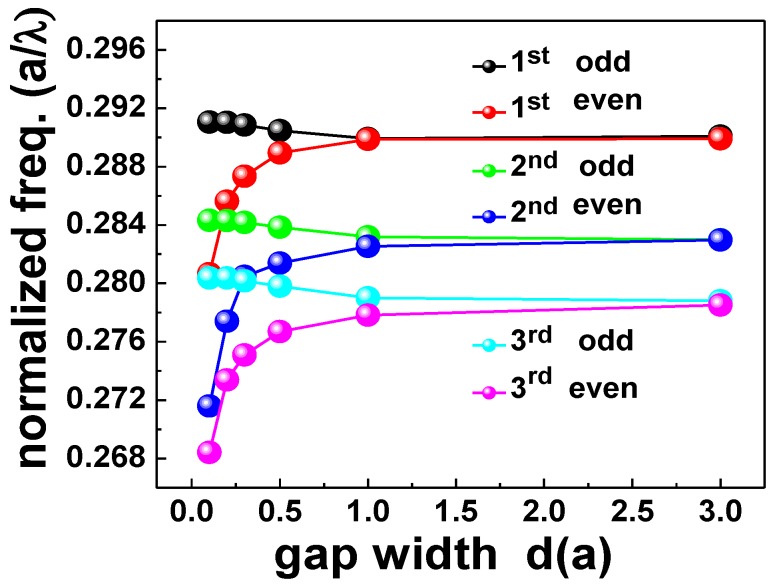
Normalized frequency at different gap width values for first, second, and third even and odd modes in the lateral coupled PhC NB cavities.

The laterally coupled PhC NB cavity structure, as shown in Figure 2a, was also numerically simulated and investigated by varying the air gap width (*d*) between the two identical PhC NB cavities. As expected from the coupled-mode theory [31], and as it is shown from mode profiles in Figure 2, the evanescent coupling produces even and odd modes. The resonant peaks of a single PhCNB cavity split into the even and odd modes as a result of the coupling of wavefunctions derived from two separate PhCNB cavities. When the gap distance between the two nanobeams reduces, the overlap of the evanescent tails of wavefunctions becomes stronger, which leads to larger splitting, as shown in Figure 4. In addition, the resonance frequency of even modes change more rapidly with the gap width variation compared to odd modes. However, different modes have different frequency-splitting responses that depend on the coupling strength and the mode order. For sensing enhancement, due to the evanescent coupling between the PhCNB cavities, light is confined in the air gap for even modes as shown in Figure 2, which leads to an enhancement of the overlapping area of the existing field with the surrounding medium. 

**Figure 5:**
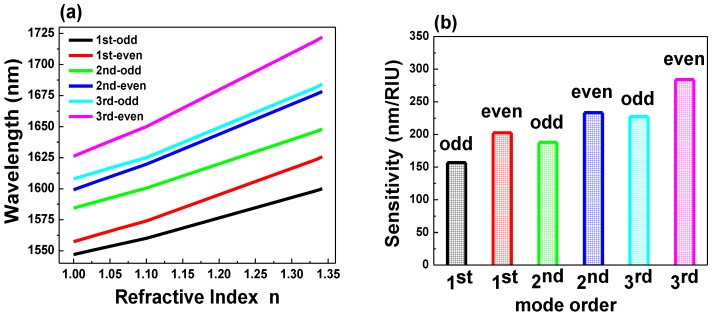
(**a**) Refractive index n *versus* wavelength shift (Δλ); (**b**) the sensitivity (S = Δλ/Δ*n*) of even and odd modes in the laterally-coupled PhC NB cavity biosensor.

Varying the refractive index of the surrounding medium of the coupled PhC NB cavity allowed us to evaluate the resonant wavelength peak shift (Δλ) of even and odd modes derived from the first three order modes (first, second, and third), as shown in Figure 5a. The slope (Δλ/Δ*n*) of each curve in Figure 5a represents the sensitivity to refractive index changes, as shown in Figure 5b. For sensing enhancement, it appears that even modes are substantially better than odd modes because light is localized in the air gap. In addition, high-order modes yield higher sensitivity because of the area of overlapping with surrounding medium is larger, which is consistent with previous results that represent a waveguide mode that has low Q-factor [43].

**Figure 6:**
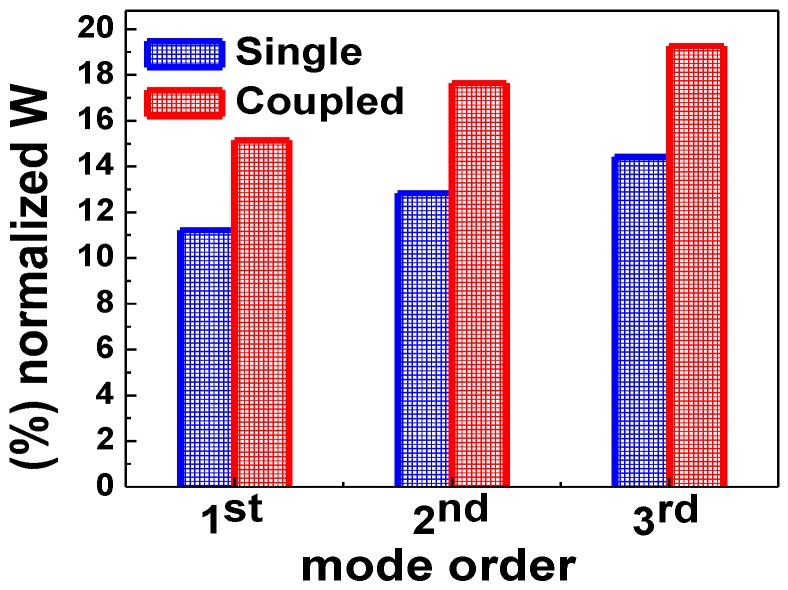
Normalized electric field energy percentage in air for the single and laterally-coupled PhC NB cavities.

From the results, which are shown in Figure 3 and Figure 5, we conclude that even modes resulting from the coupling between PhCNB cavities have higher sensitivity compared to the single PhC NB cavity, especially for high-order modes. To confirm this result, electric field energy (W) was calculated for both single and laterally coupled PhC NB cavities. The calculation of electric field energy in air, which is calculated according to Equation (1) [44] and is shown in Figure 6, also shows that higher order even modes have higher sensitivity. Here, the electric field energy in Figure 6 is normalized to the total electric field energy in the space domain:

(1) $$W=\frac{\text{1}}{\text{2}}\int E^{\text{*}}•D\;\text{dv}$$

where [(E* • **D**)/2] represents the electric field energy density. Figure 6 shows a comparison of the energy percentage that extends from the dielectric to air space and overlaps with the surrounding medium refractive index for single and laterally-coupled PhC NB cavities. For laterally-coupled PhC NB cavities, the amount of energy is higher than the single PhC NB cavity due to the evanescent coupling between the two identical PhC NB cavities. Hence, it explains the higher sensitivity of the laterally-coupled PhC NB cavities compared to the single PhC NB cavity. If the electromagnetic field could be pushed into the low index material and over a large area of the surface that means a good chance for more overlapping between the electromagnetic field and the detected material. As a result, the resonance peak wavelength will be very sensitive to any variation in the surrounding medium refractive index. 

Table 1 shows a comparison between the single PhC NB and laterally coupled PhC NB cavities for various modes (1st, 2nd, and 3rd). In addition, it shows that even modes, high order modes, and the coupled PhC NB cavities have higher sensitivity. These findings could serve as a useful guide toward a good design of label-free optical biosensor.

**Table 1:** A comparison between the single PhC NB cavity and the laterally-coupled PhC NB cavities (even modes).

Structure	Single PhC NB			Coupled PhC NB (Even Modes)		
Mode order	1st	2nd	3rd	1st	2nd	3rd
Sensitivity (nm/RIU)	176	215	240	203	234	284
Normalized Energy in air (%)	11.2	12.8	14.4	15.2	17.6	19.4
Normalized frequency (a/λ) of the fundamental mode	0.2900 (Cal. & Exp.)			0.28896 (Cal. & Exp. at gap width *d* = 0.5*a*)		

## 3. Fabrication and Optical Characterization

**Figure 7:**
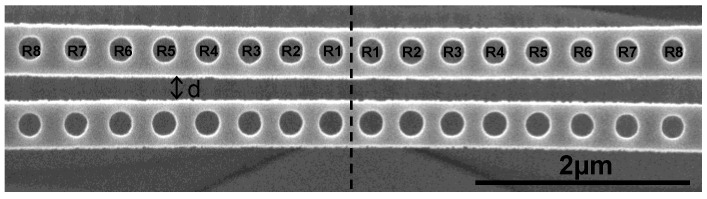
SEM image of a laterally-coupled PhC NB cavity with lattice constant (*a* = 450 nm) and gap distant (*d* = 0.5*a*).

We fabricated the laterally coupled PhC NB cavities by combining two identical PhC NB cavities made from a 240 nm thick InGaAsP layer on the InP substrate. The InGaAsP layer consists of four 10 nm thick strained InGaAsP quantum wells (QWs) which is designed for lasers operating near the 1550 nm wavelength. First, a silicon nitride (SiNx) layer and a polymethylmethacrylate (PMMA) layer were deposited for the dry etching processes and electron beam lithography. Then the patterns of PhCNB cavities were defined by electron beam lithography followed by two dry etching procedures in the inductive couple plasma (ICP) system. At the end, the InP substrate was removed by HCl solution. Figure 7 shows the SEM image of a fabricated laterally coupled PhC NB cavity biosensor which has a lattice constant (*a* = 450 nm) and a gap width (*d* = 0.5*a*). The fabricated devices were characterized at room temperature via optical pumping by an 850 nm pulsed laser with pulse width of 30 *ns* and duty cycle 1.5%. The pumped spot was focused with a 100× objective lens. Next, the emitted light was collected by an objective lens coupled to a multimode fiber which connects to an optical spectrum analyzer (OSA). More than ten sets of fabricated devices with different air-slot gap width values were measured.

**Figure 8:**
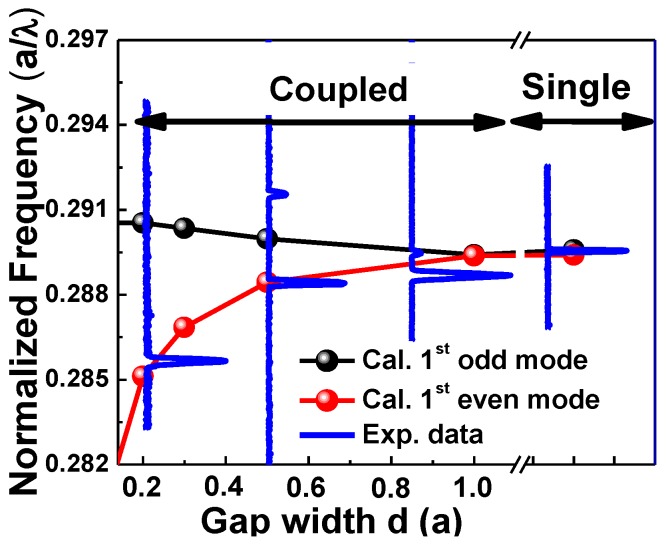
(**blue**) Lasing spectra of the single and laterally-coupled PhC NB cavities with gap width (0.8*a*, 0.5*a* and 0.26*a*). (**black and red**) the normalized frequencies of first-order odd/even modes from the numerical calculation.

Figure 8 shows the lasing spectra of both the single and laterally-coupled PhCNB cavities with gap widths (0.8*a*, 0.5*a*, and 0.26*a*). In Figure 8, the black and red curves represent the simulated first-order odd and even modes, respectively, and the blue curves represent the observed lasing spectra. Obviously, the lasing frequency splits up while the gap width reduces that is consistent with our simulation results in Figure 4.

**Figure 9:**
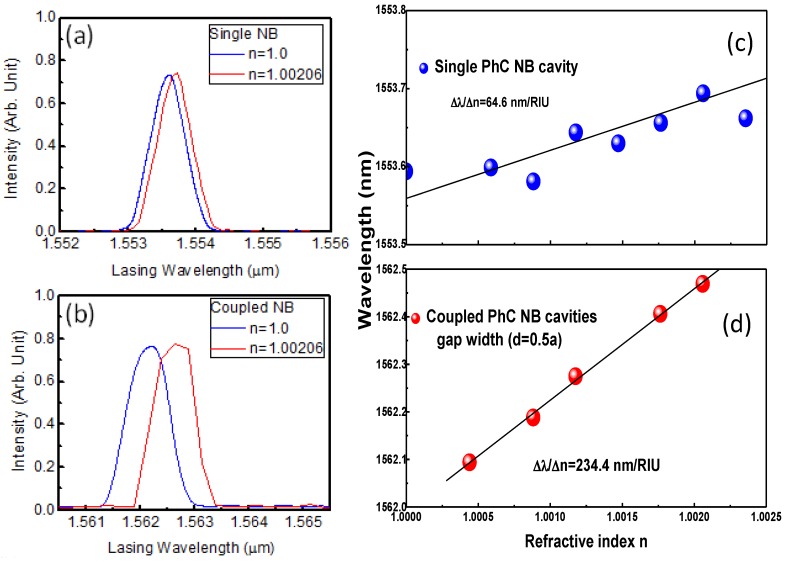
The measured lasing spectra from (**a**) the single NB cavity and (**b**) the coupled NB cavity with refractive indices of 1.0 (blue) and 1.00206 (red). Lasing wavelengths correspond to refractive index variation. The blue and red dots represent (**c**) single and (**d**) laterally-coupled PhC NB cavity with gap distance 0.5a.

In order to observe the lasing action with the refractive index variation of surrounding medium, the fabricated devices were placed into a gas chamber filled with CO_2_ gas. In this chamber, the surrounding medium’s refractive index can be controlled by changing the density of CO_2_ gas. After that, these devices were optically pumped. Then, the emission was collected within the gas chamber while increasing the density of CO_2_ gas in the chamber. The relation between the refractive index and gas pressure is given by:

(2) $$n_{\text{tp}}-\text{1}=\text{(}n_{\text{s}}-\text{1)}\times \frac{p\text{[1}+p\text{(60.1}-\text{0.972}t\text{)}\times {\text{10}}^{-\text{10}}\text{]}}{\text{96095.43(1}+\text{0.003661}t\text{)}}$$

where *n*_tp_, *n*_s_, *t*, and *p* denote the refractive index under this surrounding condition, refractive index at 150C and 1 atm, temperature in Kelvin, and pressure in Pa, respectively. Figure 9a,b shows the measured lasing spectra from the single NB cavity and the coupled NB cavity with the environmental refractive indices of 1.0 (blue) and 1.00206 (red). Figure 9c,d is the lasing wavelengths at different surrounding medium index of the single and lateral coupled PhC NB cavity with a gap width (*d* = 0.5*a*). The sensitivity (S = ∆λ/∆*n*) of the single PhCNB laser was 65 nm/RIU, while for the laterally-coupled PhCNB laser was 234 nm/RIU. In addition, the observed line-widths of these two lasers were 0.45 nm and 0.63 nm, respectively. To evaluate the biosensor performance, we evaluated the FOM as defined in reference [28]:

(3) $$\text{FOM}=\frac{\text{Δλ/Δ}n}{\text{δλ}}$$

where δλ is the line-width of the resonance peak. Higher FOM of the sensor means a good label-free biosensor performance. By using Equation (3), in addition to the experimental data, we evaluated the FOM of the single PhCNB laser, as 145, and FOM of the lateral coupled PhC NB laser with gap width (*d* = 0.5*a*) as 372. Therefore, the performance of the coupled PhC NB laser is about 2 to 3 times higher than the single PhC NB cavity. For sensing, the laterally-coupled PhC NB cavity, as a biosensor, is much better than the single PhC NB cavity. In sensing, the ability to detect a small change of the surrounding medium’s refractive index is limited by the line-width (Q-factor) of the cavity. A higher Q-factor means it is easier to detect a very small shift in the resonance that enhances the sensor performance. The Q-factor of the resonator can be significantly enhanced by optimizing the device geometry. In Table 2, we show the experimental results of sensitivity and line-width for various PhC structures of previous works compared to our experimental data. Our experimental results clearly show that the laterally-coupled PhC NB cavity biosensor has narrower line-width (higher Q-factor) compared to similar sensors [43], even though some of them have higher sensitivity.

**Table 2:** Sensitivity, line-width and figure of merit (FOM) of various optical structures compared to our experimental results.

Structure	Sensitivity S(Δλ/Δn) [nm.RIU^−1^]	Line-Width (δλ) [nm]	FOM (S/δλ)
PhC waveguide [45]	88		
1D-PhC cavity array [46]	130	~0.78	~166
Ho-cavity array [23]	115	~0.78	~147
1D-PhC array [47]	480	~10.33	~46.4
2D-PhC [48]	510	10.0	51
Slot PhC NB waveguides [43]	700	~2.8	~250
Slotted 2D-PhC [15]	1538	~0.41	~3751.2
Hybrid resonator [40]	120		
Microring resonator [40]	65		
Single PhCNB	65	~0.45	~145
Coupled PhCNB	234	~0.63	~372

In addition to the small size of the proposed structure, which is wavelength scale, and its high Q-factor which improves the figure of merit and detection resolution. The properties of this coupled PhCNB cavities can be tuned and optimized by adjusting the gap width, especially for even modes because, as shown in the numerical modeling of the field distribution, the field is very strong in the gap outside of the cavity structure due to the strong coupling between the two cavities. Additionally, this proposed structure could be a good candidate for a high-throughput and multi-target detection chip based on an array of PhCNB cavities. Finally, trade-off between sensitivity, Q-factor for a good biosensor configuration is required for a specific biosensing application.

## 4. Conclusions

High Q-factor and high FOM are related and they lead to high-performance label-free optical biosensors. The laterally-coupled PhC NB cavity structure was investigated numerically by 3D-FDTD simulation and experimentally as a label-free biosensor. By using the design of an air-slot PhC cavity, the electromagnetic field is confined in the air or liquid instead of the dielectric material, which increased the sensitivity compared to the single PhC NB cavity. The gap width between the laterally-coupled PhC NB cavities significantly affects the coupling between the two cavity modes, which causes a frequency splitting between the even and odd modes. Even modes and high-order modes are found to be more sensitive to the variation of surrounding refractive index. The high Q-factor, FOM, small mode volume, and small footprint of the sensing part make this structure a promising candidate for lab-on-a-chip integrated label-free biosensors.
